# Microfragmented Adipose Tissue Injection (MFAT) May Be a Solution to the Rationing of Total Knee Replacement: A Prospective, Gender-Bias Mitigated, Reproducible Analysis at Two Years

**DOI:** 10.1155/2021/9921015

**Published:** 2021-06-09

**Authors:** Nima Heidari, Tiffanie-Marie Borg, Stefano Olgiati, Mark Slevin, Alessandro Danovi, Brady Fish, Adrian Wilson, Ali Noorani

**Affiliations:** ^1^The Regenerative Clinic, 18-22 Queen Anne Street, London, UK; ^2^next AI, London, UK; ^3^Department of Life Sciences, Manchester Metropolitan University, UK; ^4^Academic Plastic and Reconstructive Surgery Group, Barts and the London School of Medicine, London, UK; ^5^Dept. of Morphology, Surgery and Experimental Medicine, Faculty of Medicine, University of Ferrara, 44121 Ferrara, Italy; ^6^Department of Quantitative Methods, University of Bergamo, 24129 Bergamo, Italy

## Abstract

**Background:**

Knee osteoarthritis (KOA) is a significant cause of disability in a globally ageing population. Knee replacement surgery has been shown to improve function and quality of life. Access to this intervention can be limited for a number of reasons including rationing of care, lack of healthcare provision in austere environments, and more recently, due to the cessation of elective orthopaedic care as a result of the COVID pandemic. Referral for knee replacement surgery is often guided by the patient's Oxford Knee Score (OKS). Recent therapies including treatment with microfragmented adipose tissue (MFAT) have emerged as alternatives to relieve pain and improve function in such patients.

**Method:**

We identified all patients with KOA Kellgren-Lawrence grade 3 and 4 in our dataset of patients treated with a single injection of MFAT and applied published OKS thresholds for referral for TKR to separate them into 3 cohorts according to their functional impairment. 220 patients (95 females, 125 males) with KOA were given one MFAT injection. The function (OKS) and quality of life (EuroQol-5) prior to and 24 months after therapy were compared.

**Results:**

MFAT injection provided a statistically significant improvement in the quality of life (EQ-5D) at 24 months in patients with a baseline OKS of 39 or less (*p* value: <0.001) as well as those with OKS of 27 or less who are deemed suitable for a knee replacement (*p* value: <0.001).

**Conclusion:**

MFAT injection improves quality of life in patients with KOA who are deemed suitable for the knee replacement. MFAT is a low-morbidity alternative biological treatment and can delay the need for total knee replacement in suitable patients.

## 1. Introduction

Knee osteoarthritis (KOA) has been ranked as the 11th highest contributor to global disability with a prevalence of 3.8% (95% uncertainty interval (UI) 3.6% to 4.1%) [1]. In an increasingly ageing population with a rise in rates of obesity, KOA is set to affect up to 15.7% of the world's population by 2032, representing a significant cause of disability, globally [2].

Since 2015, over 100,000 knee replacements have been performed in the UK annually [3]. This procedure has been shown to improve quality of life and be cost effective [4]. NICE guidance suggests the referral of patients to secondary care if noninterventional means have failed to relive pain of KOA [5]. In the setting of secondary care, shared decision making is undertaken between an orthopaedic surgeon and the patients to balance the risks and benefits of arthroplasty. Many clinical commissioning groups have started to use the Oxford Knee Score (OKS) as a screening tool for selecting patients for referral [6]. Although evidence behind this approach is still weak, it is likely to continue and become refined to address many of the criticisms against it [7]. A recent study has validated a model to link an individual's preoperative Oxford score to their probability of a meaningful improvement after arthroplasty and determined the effect of setting different and varying preoperative scores as thresholds for referral into secondary care [8, 9]. By setting a threshold of 31 points on the OKS, they estimated that patients with a ≥70% chance of improvement would be identified. Thresholds of 25 and 36 points would identify patients with 80% and 50% chance of meaningful improvement, respectively [8]. However, by restricting access to knee replacement surgery to those with an OKS of <27 some 10% would be denied the procedure [10].

Recently, new therapies have emerged as a possible alternative to knee replacement for relieving the pain of KOA [11–15]. We have created a model based on data from the treatment of knee OA with microfragmented adipose tissue (MFAT) [11] that allows the identification of individual who can benefit from this treatment with meaningful improvement of ≥7 in in the OKS [16] at 1 and 2 years. This can lead to the relief of pain from KOA and a delay in the need for TKR, thus providing an alternative for TKR in a selected group of patients. In this study, we report the improvement in quality of life (EQ-5D) according to a number of variables prior to treatment (baseline) with an intra-articular injection of MFAT.

We hypothesize that there will be no detectable improvement in the quality of life at 2 years following a single injection of MFAT for the treatment of KOA.

## 2. Method

The study was conducted in accordance with the principles of Good Clinical Practice (NIHR) and the General Medical Council (GMC) guidelines on research, patient consent to research, and future publication, as well as adhering to and in accordance with the Declaration of Helsinki.

### 2.1. Patient and Public Involvement Statement

The study was carried out in a private practice setting. Patient participants were not involved in the design, conduct, or plans of this analysis.

#### 2.1.1. Dataset

From 2017 to 2019, we treated 344 knees in patients with Kellgren-Lawrence grade 3 and 4 KOA (150 females and 194 males) who come to our clinic with a single injection of MFAT. All patients were reviewed by an orthopaedic surgeon and were informed of all possible options for treating their KOA including conservative means as well as injections of a number of substances including steroids, hyaluronic acid, platelet rich plasma, and microfragmented adipose tissue. They also had surgical options detailed to them including osteotomy and partial and total knee replacement where appropriate.

Following informed consent, they were offered an injection of MFAT if they fulfilled the following criteria: aetiology of KOA confirmed as idiopathic, no deformity greater than ten degrees of the varus or valgus, and the presence of KOA as diagnosed on X-ray and/or MRI. Exclusion criteria included recent injury (<3 months) of the symptomatic knee, infectious joint disease, malignancy, pregnancy, anticoagulation or thrombocytopenia, coagulation disorder, and intraarticular steroid injections performed within the last three months.

Outcomes were measured using the Oxford Knee Score (OKS) for function and the EuroQol-5 Dimension (EQ-5D) for quality of life. All patients completed these questionnaires before treatment, and at three months, six months, one year, and two years following treatment. The analysis in this report includes the 24 months' data.

OKS [17] comprises 12 questions that are scored 0-4 with 0 being severe compromise and 4 being no compromise, covering pain and function of the knee (Supplementary File 1). The best outcome is a score of 48, and the worst score possible is 0. This is a validated score for the measure of functional outcomes in patients undergoing knee arthroplasty.

EQ-5D [18, 19] is a standardized instrument developed by the EuroQol Group in order to measure the health-related quality of life in a wide range of medical conditions. Five dimensions are measured in the respondent: mobility, self-care, usual activity, pain, and anxiety/depression. Scores were given between 1 and -1, with 1 being associated with a better quality of life whilst -1 the opposite.

Other parameters that were recorded include gender, age at time of operation, and BMI (kg/m^2^).

#### 2.1.2. Setting of OKS Threshold

We set the upper threshold for analysis at OKS of >39. This has been shown to be the upper limit of preoperative score where patients can still have a clinically meaningful benefit from knee arthroplasty [8].

We set the second threshold for analysis at >27 OKS ≤ 39. By setting the threshold for offering TKR at an OKS ≤ 27, some 10% of those who would benefit from this intervention would miss out. This 10% of all those who would benefit are all in the group >27 OKS ≤ 39 [10].

We set the third threshold on the OKS ≤ 27 points where patients have a ≥70% chance of improvement with a knee replacement [8]. In this analysis, we can assess the likely benefit of an intra-articular injection of MFAT for patients who are very likely to have an improvement following a TKR [10].

These thresholds have been utilised to group the patients into 3 cohorts. The change in quality of life (EQ-5D) has been assessed for each cohort.

#### 2.1.3. Gender-Specific Analysis

All analysis has been carried out separately on male and female cohorts as we have noted a difference in the response to MFAT injection between men and women [11].

#### 2.1.4. Other Parameters

We also report the change in functional scores (OKS) in each of the three cohorts described above as well as with respect to age at baseline, KL grades 3 and 4, and BMI.

#### 2.1.5. Statistical Analysis

By study design, the paired samples have been selected and not randomized; thus, we did not assume a Gaussian distribution. Hence, the variation in the scores has been analysed utilizing a nonparametric paired sample Wilcoxon test to assess statistically significant changes in the EQ-5D scores before and after treatment at 24 months. For the same reason, summary statistics report median and interquartile ranges (IQR).

Summary statistics, statistical analysis, and statistical significance testing are reproducible with open access statistical software R (version 4.0.0 or higher; R function Wilcox test). All figures have been generated automatically from data by open access statistical software R (version 4.0.0 or higher; libraries ggpubr and PairedData).

### 2.2. Harvesting Adipose Tissue and Injecting MFAT

Full method for this technique has previously been described [20]. This injection is carried out as a day case procedure under sedation. To harvest the adipose tissue, a 17G blunt cannula connected to a Luer-lock 60 cc syringe is inserted through a small incision into the subcutaneous fat of the lower abdominal layer enabling injection of 50 ml aliquots of 150-200 ml Klein sterile solution (lignocaine, adrenaline, and 0.9% sodium chloride). A 13G blunt cannula connected to a Vaclock 20 ml syringe is then used to manually harvest approximately 50 ml adipose tissue.

The lipoaspirate is processed using a single-use device (Lipogems® system) that fragments the fat mechanically, removes impurities (e.g., blood and debris), and filters the product over 20 minutes. The resulting solution contained approximately 33% pericytes representing approximately 9 × 10^3^per ml MFAT with 1 : 100 cells from raw adipose tissue being stem cells [21] compared with only 1 : 100,000 when derived from the bone marrow [22]. Using ultrasound guidance, 6-8 ml of the resulting MFAT is then injected directly into the suprapatellar pouch of the knee joint, just proximal to the patella.

## 3. Results

A total of 220 patients (95 females and 125 males) were treated for KOA with a single injection of MFAT. This translated to 344 knees being treated, 124 patients having both knees and 96 just having one side treated (Table 1).

**Figure 1:**
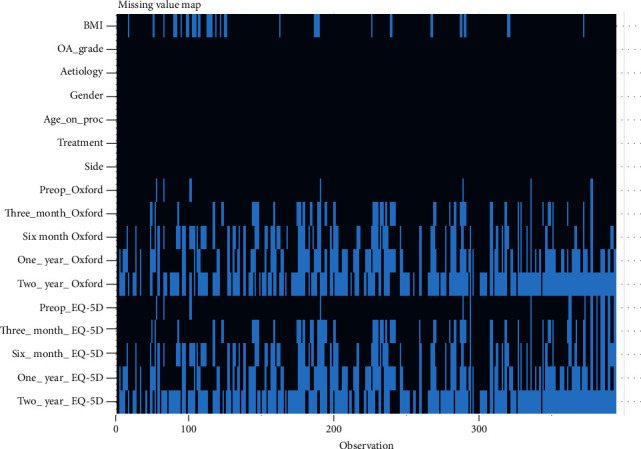
Missingness map of the data *x*-axis: outcome variables: Kellgren-Lawrence KOA grade (OA grade), aetiology of KOA, gender, age at the time of the procedure, body mass index (BMI), Oxford Knee Score (OKS) for function, and EQ-5D for quality of life at preoperative baseline and three- and six-month and one- and two-year follow-up. *y*-axis: data points missing (21%) due to patients lost to follow-up (white).

The missing values from the dataset are shown in Figure 1. Missingness refers to a value not being recorded for a variable due to patients being lost to follow-up. Data points are missing in 25% of instances due to patients lost to follow-up.

### 3.1. Quality of Life (EQ-5D) within Each of the Strata of Functional Outcomes (OKS)

Following the stratification of patients according to their baseline OKS, the EQ-5D at baseline and 2 years postprocedure were compared. The data for females and males has been analysed separately.

**Figure 2:**
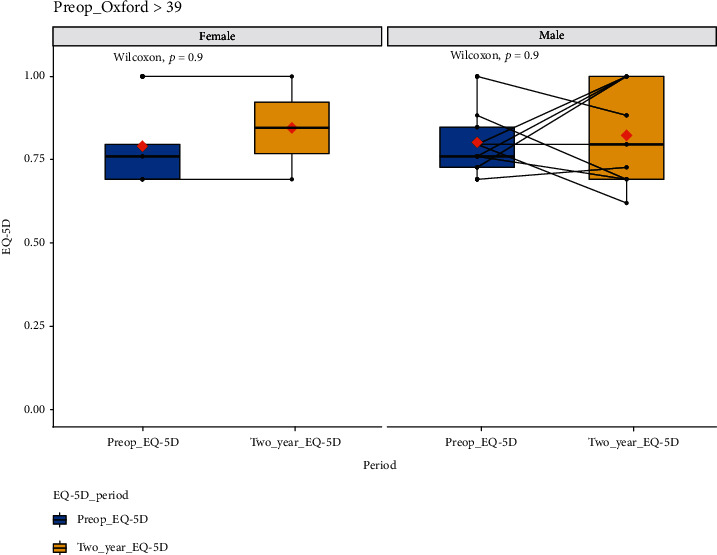
EQ-5D scores at baseline and 2-year follow-up for all those with OKS > 39. Male and female data have been plotted separately. *x*-axis: EQ-5D for the quality-of-life score at preoperative baseline and at two-year follow-up. *y*-axis: EQ-5D for the quality-of-life score value; boxplot showing *L*-estimators: outliers plotted as individual points at 2-year follow-up. Full data tabulated in Table 1. Connecting lines: heuristic visualization of single-patient trajectories of variation of EQ-5D for the quality-of-life score value for function between preoperative baseline and two-year follow-up; plotted with R (version 4.0.0 or higher; libraries ggpubr and PairedData). Source: authors' data and reproducible statistical analysis with open access statistical software R (version 4.0.0 or higher).

Figure 2 and Table 2 demonstrate the EQ-5D boxplot and tabulated data, respectively, for 9 female and 31 male patients with an OKS of greater than 39. Although there is a general trend towards improvement of EQ-5D scores, this is not statistically significant (*p* = 0.9 for female and male patients) at 2 years following treatment.

**Figure 3:**
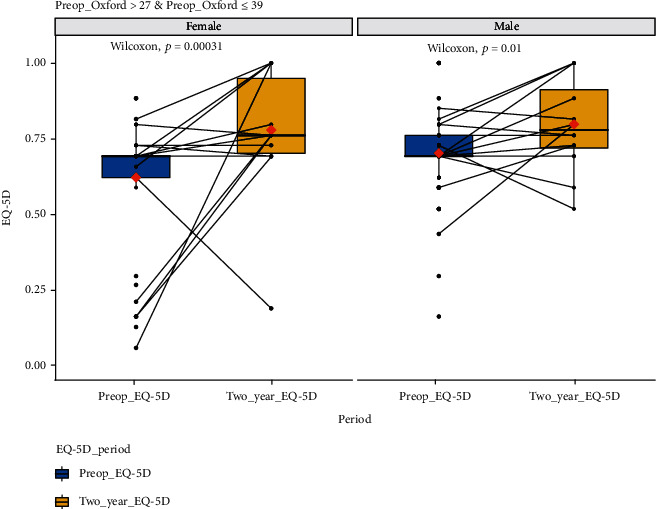
EQ-5D scores at baseline and 2-year follow-up for all those with >27 OKS ≤ 39. Male and female data have been plotted separately. *x*-axis: EQ-5D for the quality-of-life score at preoperative baseline and at two-year follow-up. *y*-axis: EQ-5D for the quality-of-life score value; boxplot showing *L*-estimators: outliers plotted as individual points at 2-year follow-up. Full data tabulated in Table 2. Connecting lines: heuristic visualization of single-patient trajectories of variation of EQ-5D for the quality-of-life score value for function between preoperative baseline and two-year follow-up; plotted with R (version 4.0.0 or higher; libraries ggpubr and PairedData). Source: authors' data and reproducible statistical analysis with open access statistical software R (version 4.0.0 or higher).

Figure 3 and Table 3 are the EQ-5D boxplot and tabulated data, respectively, for 53 female and 81 male patients with and OKS of greater than 27 but less than or equal to 39. This group had a highly statistically significant improvement in their quality of life (*p* < 0.05 for both males and females) at 2 years following treatment.

**Figure 4:**
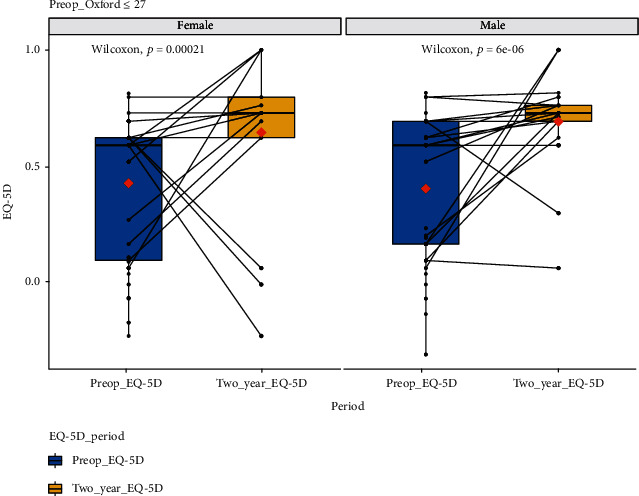
EQ-5D scores at baseline and 2-year follow-up for all those with OKS ≤ 27. Male and female data has been plotted separately. *x*-axis: EQ-5D for the quality-of-life score at preoperative baseline and at two-year follow-up. *y*-axis: EQ-5D for the quality-of-life score value; boxplot showing *L*-estimators: outliers plotted as individual points at 2-year follow-up. Full data tabulated in Table 3. Connecting lines: heuristic visualization of single-patient trajectories of variation of EQ-5D for the quality-of-life score value for function between preoperative baseline and two-year follow-up; plotted with R (version 4.0.0 or higher; libraries ggpubr and PairedData). Source: authors' data and reproducible statistical analysis with open access statistical software R (version 4.0.0 or higher).

Figure 4 and Table 4 are the EQ-5D boxplot and tabulated data, respectively, for 84 female and 77 male patients with and OKS of less than or equal to 27. This group had a highly statistically significant improvement in their quality of life (*p* < 0.05 for both females and males) at 2 years following treatment.

### 3.2. Functional Outcomes (OKS)

**Figure 5:**
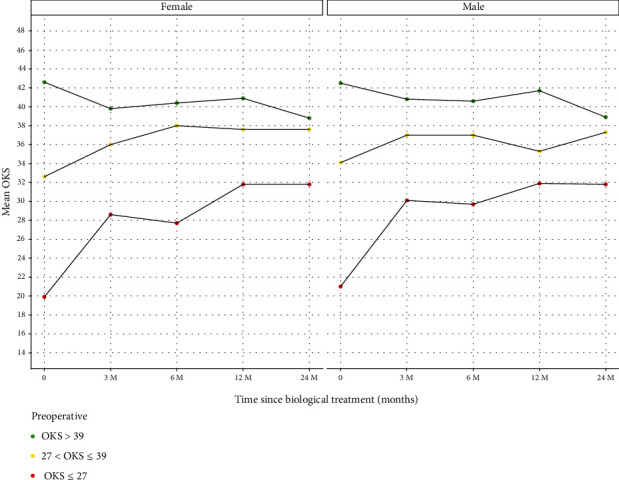
Patients grouped by OKS at baseline with follow-up OKS at 3, 6, 12, and 24 months. *x*-axis: Time points in relation to treatment: baseline prior to treatment (0), 3, 6, 12, and 24 months posttreatment. *y*-axis: OKS mean for each group. Green (d1): baseline OKS > 39. Yellow (d2): baseline > 27OKS ≤ 39. Red (d3): baseline OKS ≤ 27.

Following the application of the thresholds to the data, the trends of change on the OKS have been charted (Figure 5) and tabulated (Table 5).

Nine females with a baseline OKS > 39 had a complete dataset. The mean OKS of this group was 42.6 (SD 2.5). At 2 years, the OKS was 38.8 (SD 3.9). Thirty-one males with a baseline OKS > 39 had a complete dataset. The mean OKS of this group was 42.5 (SD 2.4). At 2 years, the OKS was 38.9 (SD 9.9).

Fifty-three females had a baseline functional score > 27 OKS ≤ 39 and a compete dataset. The mean baseline OKS for this group was 32.6 (SD 3.5). At 2 years, this improved to an OKS of 37.6 (SD 7.6). Eighty-one males had a baseline functional score > 27OKS ≤ 39 and a complete dataset. The mean baseline OKS for this group was 34.1 (SD 3.5). At 2 years, this improved to an OKS of 37.3 (SD 8.8).

Eighty-four females had a baseline OKS ≤ 27 with a complete dataset. The mean OKS for this group at baseline was 19.9 (SD 5.1). At 2 years, this improved to an OKS of 32 (SD 11.1). Seventy-seven males had a baseline OKS ≤ 27 with a complete dataset. The median OKS for this group at baseline was 21.0 (SD 5.4). At 2 years, this improved to an OKS of 31.8 (SD 10.2).

#### 3.2.1. Age

**Figure 6:**
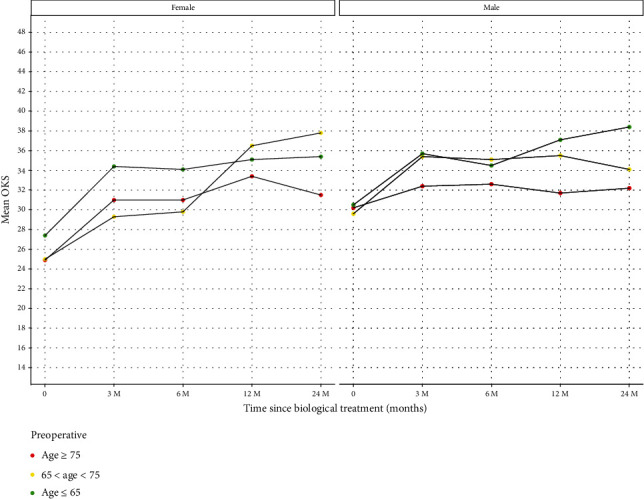
Patients grouped by age at time of treatment (baseline) with follow-up OKS at 3, 6, 12, and 24 months. *x*-axis: time points in relation to treatment: baseline prior to treatment (0), 3, 6, 12, and 24 months posttreatment. *y*-axis: OKS mean for each group. Green: age at time of treatment ≤65. Yellow: age at time of treatment 65 < age < 75. Red: age at time of treatment ≥75.

We grouped the patients into 3 age groups which included those 75 years and older, those between 65 to 75 years, and all those 65 years and younger. The trends of improvement were seen in all age groups. The summary of the scores is seen in Figure 6 and Table 6.

There were 41 females over the age of 75. The mean OKS for this group was 24.9 (SD 7.0) at baseline. This improved to 31.5 (SD 10.9) at 2 years. There were 35 males in the over 75 age group with a baseline mean OKS of 30.2 (SD 8.2) at baseline which improved to 32.2 (SD 10.7) at 2 years.

There were 48 females aged 65 to 75 with a mean OKS of 25.0 (SD 10.3) at baseline which went on to improve to 37.8 (SD 9.4) at 2 years. There were 64 males aged 65 to 75 with a mean OKS of 29.6 (SD 8.2) at baseline which went on to improve to 34.1 (SD 10.6) at 2 years.

There were 61 females in the under 65-year age group with a mean OKS of 27.4 (SD 7.9). They improved to a mean OKS of 35.4 (SD 8.1) at 2 years. There were 95 males aged below 65 with a mean OKS of 30.5 (SD 10.2) at baseline. This improved to 38.4 (SD 8.0) at 2 years.

#### 3.2.2. KOA Grade

**Figure 7:**
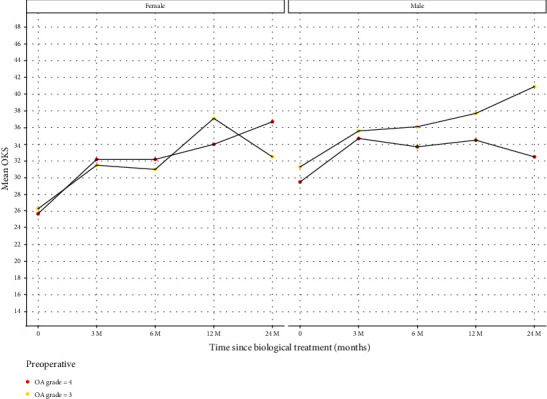
Patients grouped by KL OA grade at time of treatment (baseline) with follow-up OKS at 3, 6, 12, and 24 months. *x*-axis: time points in relation to treatment: baseline prior to treatment (0), 3, 6, 12, and 24 months posttreatment. *y*-axis: OKS mean for each group. Red (d1): KL OA grade 4 at time of treatment. Yellow (d2): KL OA grade 3 at time of treatment.

Patients were grouped by the severity of the KOA on knee radiographs. We analysed the data for Kellgren-Lawrence grades 3 and 4 separately. The summary data is charted in Figure 7 and tabulated in Table 7. The pattern of response is very similar in both genders and both grades at 12 months postinjection but there is a deterioration in females with grade 3 between 12 and 24 months and similar deterioration in the males who have grade 4 OA.

### 3.3. Body Mass Index

**Figure 8:**
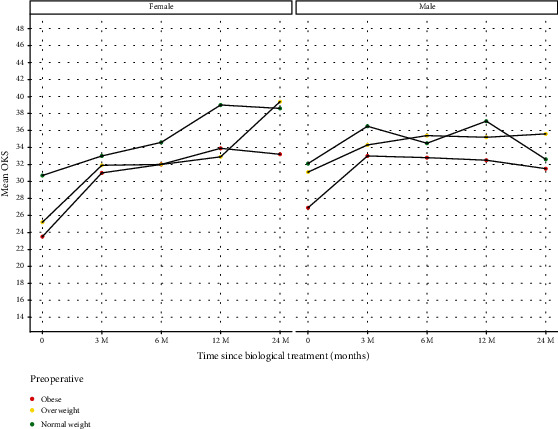
Patients grouped by BMI at time of treatment (baseline) with follow-up OKS at 3, 6, 12, and 24 months. *x*-axis: time points in relation to treatment: baseline prior to treatment (0), 3, 6, 12, and 24 months posttreatment. *y*-axis: OKS mean for each group. Red: obese (BMI ≥ 30). Yellow: overweight ≥25 BMI < 30). Green: healthy weight (BMI < 25)

Analysis of patients according to their BMI is summarised in Figure 8 and Table 8.

### 3.4. Complications

Table 9 demonstrates the complications observed in this patient cohort. The most common complication in this group was joint swelling and pain, occurring in 48 (14%) of knees. In the majority of cases, this was relieved with standard postoperative analgesia with only 14 (4%) required a modification of the standard postoperative analgesia patients were prescribed upon discharge.

Of the 9 patients with harvest site bleeding, 6 required more dressing changes and early review by our plastic and reconstructive surgeon. All went on to resolve without further consequence at final follow-up.

Of the 14 patients who had pain at the harvest site exceeding that usually expected, two required a modification of their postoperative analgesia. All went on to resolve without further consequence at final follow-up.

One patient had a severe reaction to the injection requiring an arthroscopic wash-out of the joint. No microorganisms were identified on prolonged cultures and infection as a cause was ruled out. The exact reason for this was not identified as the patient was lost to follow-up.

## 4. Discussion

We report a threshold model based on previously published work [10] for identifying individuals with KOA who may benefit from the use of MFAT to improve their quality of life. This may provide an alternative treatment for those who are unable to access knee replacement surgery. The groups of patients that we report here are those with the more severe grades of KOA (KL 3 and 4) who would be suitable for knee replacement surgery. We have found that below the threshold of OKS ≤ 39 a statistically significant improvement in quality of life is demonstrated with that with an OKS of ≤27 having the greatest benefit. The null hypothesis for this study was that MFAT would not result in a statistically significant improvement in the quality of life and knee function. Based on our results, we were able to reject our null hypothesis for the threshold groups of “OKS ≤ 27” and “>27 OKS ≤ 39” and accept our alternative hypothesis that MFAT does result in a statistically significant improvement in the knee function and quality of life. We were unable to reject our null hypothesis regarding change in quality of life in patients with an OKS ≥ 39. This includes a small group in our data with only 9 females and 31 males. It is unusual for individuals with severe KOA on imaging to have good function but the occasional disparity between imaging and function is well recognised [19].

Knee replacement surgery has been shown to improve quality of life for KOA sufferers and is cost effective [9]. However, not all may benefit from it as unfortunately up to 20% of those undergoing TKA are dissatisfied with the outcome [23, 24] due to continued pain, with a further 2–3% experiencing life-altering complications. In our patient cohort, a very low number of adverse events were recorded. The most common complication was joint swelling and pain, resulting in deviation from standard postoperative analgesia in 14 knees of the 344 treated. Other complications in our patient cohort included harvest site bleeding, pain at the harvest site, and one reaction to the injection. The safety of MFAT and the minimal long-term complication rate is also reflected in other studies. A meta-analysis of 36 clinical trials involving harvesting of mesenchymal stem cells confirmed no evidence of increased infection, acute toxicity, or organ system complications [25]. In another study by Berman et al. [26] involving vascular fraction procedures in 1524 patients, there were no complications in 98% of cases.

Our results highlight that variables such as sex, age, and BMI also influence the resulting benefit following MFAT therapy. The response between men and women varies in our results. Men with Kellgren-Lawrence grade 3 disease have a greater improvement following MFAT (Figure 7). The improvement was maximally shown in men under 65 years (Figure 6). Women of the same Kellgren-Lawrence grade 3 disease demonstrated less of a response to MFAT at 2 years. In women, age is the major influencing variable that affects the improvement in OKS following MFAT (Figure 6), with little variation demonstrated in women in relation to BMI (Figure 8). These gender differences may be due to a combination of genetic, metabolic, and endocrine factors [20].

Another well-known problem with MFAT is the poorer outcomes and higher risk of early failure of knee replacement in younger patients. For patients aged 50–54 years, the lifetime risk of revision (LTRR) is 35%, compared with the LTRR of around 5% in patients aged 70 years. Interestingly, women have an approximately 15% lower LTRR [27].

As a result of the COVID-19 pandemic, the number of knee replacement procedures carried out has dramatically reduced. This has resulted in the longest waiting list for orthopaedic surgery in living memory. Resumption of elective orthopaedic surgery will also require a change in routines and lead to a reduction of capacity [28].

This study demonstrates the benefit of MFAT administration in patients with OKS ≤ 39 in which a statistically significant improvement in quality of life, with the greatest benefit occurring in patients with an OKS of ≤27 (Figure 5). Our findings corroborate those of other investigators, including Gobbi et al. (2021), who demonstrated a clinical, functional, and quality of life importance at two years post-MFAT therapy in a multicentric international study involving elderly patients with Kellgren and Lawrence grade 2-4 [29]. Mautner et al. demonstrated that giving either MFAT or bone marrow aspirate concentrate significantly improved knee function, pain, and quality of life in patients with KOA, with no statistically significant difference in efficacy between therapies [30].

### 4.1. Strengths and Limitations

This analysis explores the quality of life before and after MFAT, taking into account patient factors such as age, sex, baseline KOA severity, and comorbidities such as high BMI. The statistical analysis of this study is reproducible with free open access software, and its results are replicable. We also detail the complications observed in our patient cohort, although the vast majority were very minor when compared to the alternative: TKA.

Limitations to this study include small numbers of patients in some cohorts. This is partially due to patients lost to follow-up, as shown by the missing data (21%) in the missingness map.

Our follow-up only reaches two years, where as many of those with TKA are followed up for much longer. It is valid to note however that the relatively short period of pain relief and functional improvement we report here is juxtaposed with the low morbidity of the MFAT injection procedure. There may even be utility in repeating the injection in order to “top-up” the effect in those who have return of symptoms at 2 years. There is a need for future investigations to address this issue.

Alternatives to total knee arthroplasty that can reduce KOA sufferers' pain and improve their function and quality of life are important to consider in these unprecedented times. There is growing evidence of the utility of biologics in treating the pain of KOA [11–14, 27, 28, 31]. In order to formally assess the benefit of MFAT in treating KOA, there needs to be further investigation into its effects by a randomised control trial. The trial will need to be appropriately powered and conducted to provide the information required to utilise this technology to its full. A detailed health economic analysis can then establish its place in the armamentarium of our healthcare system in treating KOA.

## 5. Conclusion

An intra-articular injection of MFAT improves the quality of life of patients with knee OA. This improvement is most pronounced in those with a baseline OKS < 39, especially women. The variation in response to MFAT between subcategories demonstrated is likely due to genomic, hormonal, and metabolic factors which should also be considered when treating patients with KOA.

The threshold model reported in this analysis supports the use of MFAT as an alternative for patients with knee OA who are unable or unwilling to undergo replacement surgery.

## Figures and Tables

**Table 1:** Patients with KOA receiving MFAT.

Gender	Patients	Total knees	Bilateral	Monolateral	Grade 3	Grade 4
Female	95	150	55	40	46	104
Male	125	194	69	56	67	127
Total	220	344	124	96	113	231

**Table 2:** EQ-5D score at preoperative baseline and at two-year follow-up for all patients with OKS > 39 at baseline.

	Female		Male	
OKS	>39			

*n*	9		31	

Period	Pre-Op	2 years	Pre-op	2 years

Min	0.6910	0.6910	0.6910	0.6200

1st Qu	0.6910	0.7682	0.7270	0.6910

Median	0.7600	0.8455	0.76	0.7960

Mean	0.7907	0.8455	0.8021	0.8231

3rd Qu	0.7960	0.9227	0.8480	1.0000

Max	1.0000	1.0000	1.0000	1.0000

*p* value	0.9		0.9	

**Table 3:** EQ-5D score at preoperative baseline and at two years' follow-up for all patients with OKS > 27 to ≤39 at baseline.

	Female		Male	
OKS	>27 to ≤39			

*n*	53		81	

Period	Pre-Op	2 years	Pre-op	2 years

Min	0.0550	-0.0770	0.1590	0.5160

1st Qu	0.6200	0.6910	0.6910	0.7180

Median	0.6910	0.7600	0.6910	0.7780

Mean	0.6204	0.7327	0.7013	0.7967

3rd Qu	0.6910	0.8980	0.7600	0.9123

Max	0.8830	1.0000	1.0000	1.0000

*p* value	0.00031		0.01	

**Table 4:** EQ-5D score at preoperative baseline and at two years' follow-up for all patients with OKS ≤ 27 at baseline.

	Female		Male	
OKS	≤27			

*n*	84		77	

Period	Pre-Op	2 years	Pre-op	2 years

Min	-0.2390	-0.2390	-0.3190	0.0550

1st Qu	0.0880	0.6200	0.1590	0.6910

Median	0.5870	0.7270	0.5870	0.7270

Mean	0.4222	0.6425	0.3391	0.6897

3rd Qu	0.6200	0.7960	0.6910	0.7600

Max	0.8120	1.0000	0.8140	1.0000

*p* value	0.00021		<0.0001	

**Table 5:** Tabulated data for Figure 5.

Gender	OKS period	Count	Mean	Median	SD	Data
Female	0	9	42.6	42.0	2.5	d1
Female	1	9	39.8	41.0	6.5	d1
Female	2	9	40.4	39.0	5.9	d1
Female	3	9	40.9	41.0	4.7	d1
Female	4	9	38.8	38.5	3.9	d1
Male	0	31	42.5	42.0	2.4	d1
Male	1	31	40.8	41.0	5.2	d1
Male	2	31	40.6	43.0	7.0	d1
Male	3	31	41.7	44.0	6.5	d1
Male	4	31	38.9	43.0	9.9	d1
Female	0	53	32.6	32.0	3.5	d2
Female	1	53	36.0	37.0	6.7	d2
Female	2	53	38.0	38.5	5.7	d2
Female	3	53	37.6	36.5	7.0	d2
Female	4	53	37.6	38.0	7.6	d2
Male	0	81	34.1	34.0	3.5	d2
Male	1	81	37.0	39.0	6.4	d2
Male	2	81	37.0	37.0	6.3	d2
Male	3	81	35.3	37.0	8.7	d2
Male	4	81	37.3	37.0	8.8	d2
Female	0	84	19.9	20.5	5.0	d3
Female	1	84	28.6	28.0	9.9	d3
Female	2	84	27.7	28.0	10.8	d3
Female	3	84	31.8	32.0	10.6	d3
Female	4	84	31.8	33.0	11.1	d3
Male	0	77	21.0	22.0	5.4	d3
Male	1	77	30.1	27.5	9.3	d3
Male	2	77	29.7	30.0	8.7	d3
Male	3	77	31.9	32.0	9.6	d3
Male	4	77	31.8	30.0	10.2	d3

OKS period: 0—baseline, 1—3 months postinjection, 2—6 months postinjection, 3—12 months postinjection, 4—24 months postinjection. Count: the number of patients in each group. Mean, median, and SD (standard deviation). Data: d1—baseline OKS of >39, d2—baseline OKS > 27OKS ≤ 39, d3—baseline OKS ≤ 27.

**Table 6:** Patients grouped by age at time of treatment (baseline) with follow-up OKS at 3, 6, 12, and 24 months.

Gender	OKS period	Count	Mean	Median	SD	Data
Female	0	41	24.9	24.5	7.0	d1
Female	1	41	31.0	32.5	9.6	d1
Female	2	41	31.0	33.0	9.0	d1
Female	3	41	33.4	34.0	9.7	d1
Female	4	41	31.5	34.0	10.9	d1
Male	0	35	30.2	31.5	8.2	d1
Male	1	35	32.4	35.0	7.4	d1
Male	2	35	32.6	32.5	7.3	d1
Male	3	35	31.7	30.0	9.1	d1
Male	4	35	32.2	35.0	10.7	d1
Female	0	48	25.0	24.0	10.3	d2
Female	1	48	29.3	29.0	10.4	d2
Female	2	48	29.8	32.5	11.0	d2
Female	3	48	36.5	36.0	7.8	d2
Female	4	48	37.8	40.0	9.4	d2
Male	0	64	29.6	29.0	8.2	d2
Male	1	64	35.4	37.0	8.7	d2
Male	2	64	35.1	37.0	8.1	d2
Male	3	64	35.5	37.0	8.8	d2
Male	4	64	34.1	36.0	10.6	d2
Female	0	61	27.4	28.0	7.9	d3
Female	1	61	34.4	35.0	8.5	d3
Female	2	61	34.1	37.5	11.0	d3
Female	3	61	35.1	37.0	10.0	d3
Female	4	61	35.4	36.0	8.1	d3
Male	0	95	30.5	32.0	10.2	d3
Male	1	95	35.7	38.0	8.8	d3
Male	2	95	34.5	37.0	9.6	d3
Male	3	95	37.1	39.5	9.3	d3
Male	4	95	38.4	39.5	8.0	d3

OKS period: 0—baseline, 1—3 months postinjection, 2—6 months postinjection, 3—12 months postinjection, 4—24 months postinjection. Count: the number of patients in each group. Mean, median, and SD (standard deviation). Data: d1—age at time of treatment ≥75, d2—age at time of treatment 65 < age < 75, d3—age at time of treatment ≤65.

**Table 7:** Patients grouped by KL OA grade at time of treatment (baseline) with follow-up OKS at 3, 6, 12, and 24 months.

Gender	OKS period	Count	Mean	Median	SD	Data
Female	0	104	25.7	25.5	7.5	d1
Female	1	104	32.2	32.0	9.3	d1
Female	2	104	32.2	33.5	9.3	d1
Female	3	104	34.0	34.0	9.1	d1
Female	4	104	36.7	38.0	7.1	d1
Male	0	127	29.5	29.0	8.4	d1
Male	1	127	34.7	37.0	8.5	d1
Male	2	127	33.7	33.0	8.2	d1
Male	3	127	34.5	37.0	10.1	d1
Male	4	127	32.5	34.0	10.6	d1
Female	0	46	26.3	24.5	10.5	d2
Female	1	46	31.5	34.0	10.3	d2
Female	2	46	31.0	35.0	13.0	d2
Female	3	46	37.1	40.0	9.7	d2
Female	4	46	32.5	36.0	12.3	d2
Male	0	67	31.3	33.0	10.6	d2
Male	1	67	35.6	38.0	8.8	d2
Male	2	67	36.1	38.5	9.6	d2
Male	3	67	37.7	37.0	6.8	d2
Male	4	67	40.9	42.0	4.6	d2

OKS period: 0—baseline, 1—3 months postinjection, 2—6 months postinjection, 3—12 months postinjection, 4—24 months postinjection. Count: the number of patients in each group. Mean, median, and SD (standard deviation). Data: d1—KL OA grade 4 at the time of treatment, d2—KL OA grade 3 at the time of treatment.

**Table 8:** Patients grouped by BMI at time of treatment (baseline) with follow-up OKS at 3, 6, 12, and 24 months.

Gender	OKS period	Count	Mean	Median	SD	Data
Female	0	43	23.5	23.0	7.3	d1
Female	1	43	31.0	30.0	10.2	d1
Female	2	43	32.0	33.5	10.8	d1
Female	3	43	33.9	33.0	8.7	d1
Female	4	43	33.2	38.5	11.1	d1
Male	0	53	26.9	27.0	10.5	d1
Male	1	53	33.0	34.0	9.3	d1
Male	2	53	32.8	33.0	10.2	d1
Male	3	53	32.5	34.5	11.1	d1
Male	4	53	31.5	30.0	10.5	d1
Female	0	42	25.2	26.5	8.7	d2
Female	1	42	31.9	35.0	10.8	d2
Female	2	42	32.0	35.0	9.6	d2
Female	3	42	32.9	34.0	11.3	d2
Female	4	42	39.4	40.0	5.8	d2
Male	0	67	31.1	32.0	8.0	d2
Male	1	67	34.3	36.0	7.9	d2
Male	2	67	35.4	37.0	7.5	d2
Male	3	67	35.2	36.0	8.7	d2
Male	4	67	35.6	36.5	8.0	d2
Female	0	45	30.7	30.5	8.3	d3
Female	1	45	33.0	33.0	9.3	d3
Female	2	45	34.6	36.0	8.8	d3
Female	3	45	39.0	41.0	6.7	d3
Female	4	45	38.6	37.0	5.4	d3
Male	0	53	32.1	32.0	9.0	d3
Male	1	53	36.5	38.0	8.2	d3
Male	2	53	34.5	34.0	8.7	d3
Male	3	53	37.1	38.0	8.1	d3
Male	4	53	32.6	36.5	12.4	d3

OKS period: 0—baseline, 1—3 months postinjection, 2—6 months postinjection, 3—12 months postinjection, 4—24 months postinjection. Count: the number of patients in each group. Mean, median, and SD (standard deviation). Data: d1—obese (BMI ≥ 30), d2—overweight (≥25 BMI < 30), d3—healthy weight (BMI < 25).

**Table 9:** Complications associated with intra-articular injection of the knee with MFAT.

Complication	No. of pts, *n* (%)	Deviation from standard post-op care, *n* (%)
Joint swelling and pain	48 (14)	14 (4)
Harvest site bleeding	9 (4)	6 (2.7)
Pain at harvest site	14 (6)	2 (0.9)
Joint washout	1 (0.5)	1 (0.5)

## Data Availability

Extra data is available by emailing n.heidari@gmail.com.

## Additional Points

*Article Summary*. (i) This study evaluates the benefit to quality of life and knee function following injection of microfragmented adipose tissue (MFAT). (ii) A statistically significant improvement in quality of life was demonstrated following MFAT injection. (iii) Assessment of the patients' Oxford Knee Score (OKS) prior to MFAT injection highlighted a threshold level at which MFAT therapy is the most beneficial. Patients with a baseline OKS of 39 or less demonstrated the greatest improvement in quality of life with MFAT injection. (iv) Strengths of this study: the quality of life before and after MFAT are explored in this analysis, with consideration to patient age, sex, baseline arthritis severity, and comorbidities. (v) Limitations of this study: there are small numbers of patients in some cohorts, partially due to patient loss to follow-up.

## Supplementary Materials

Supplementary Materials This manuscript has one supplementary file that details the Oxford Knee Scoring system. This is a tool used to determine the severity of knee arthritis. A score of 0-19 may indicate severe arthritis that is likely to necessitate surgical intervention, a score of 20-29 suggests moderate to severe arthritis, a score of 30-39 suggests mild to moderate arthritis, and a score of 40 or more suggests a normally functioning knee joint.
