# Supplementary material for: Microfragmented Adipose Tissue Injection (MFAT) May Be a Solution to the Rationing of Total Knee Replacement: A Prospective, Gender-Bias Mitigated, Reproducible Analysis at Two Years
Source: Stem Cells Int. 2021 Jun 9;2021:9921015. doi: 10.1155/2021/9921015 (PMC8211497; doi:10.1155/2021/9921015)
Supplement: Supplementary Materials — This manuscript has one supplementary file that details the Oxford Knee Scoring system. This is a tool used to determine the severity of knee arthritis. A score of 0-19 may indicate severe arthritis that is likely to necessitate surgical intervention, a score of 20-29 suggests moderate to severe arthritis, a score of 30-39 suggests mild to moderate arthritis, and a score of 40 or more suggests a normally functioning knee joint. [file 9921015.f1.docx]

**Supplementary File 1 – Oxford Knee Score:**

| **Question** | **Response** |
| --- | --- |
| 1. During the past 4 weeks how would you describe the pain you usually have from your knee? | - None - Very mild - Mild - Moderate - Severe |
| 1. During the past 4 weeks have you had any trouble with washing and drying yourself (all over) because of your knee? | - No trouble - Very little trouble - Moderate trouble - Extreme difficulty - Impossible to do |
| 1. During the past 4 weeks have you had any trouble getting in and out of a car or using public transport because of your knee? (whichever you tend to use) | - No trouble - Very little trouble - Moderate trouble - Extreme difficulty - Impossible to do |
| 1. During the past 4 weeks for how long have you been able to walk before pain from your knee becomes severe? (with or without a stick) | - No pain/more than 30 minutes - 16-30 minutes - 5-15 minutes - Around the house only - Not at all – pain severe when walking |
| 1. During the past 4 weeks after a meal (sat at a table), how painful has it been for you to stand up from a chair because of your knee? | - Not at all painful - Slightly painful - Moderately painful - Very painful - Unbearable |
| 1. During the past 4 weeks have you been limping when walking because of your knee? | - Rarely/never - Sometimes, or just at first - Often, not just at first - Most of the time - All of the time |
| 1. During the past 4 weeks could you kneel down and get up again afterwards? | - Yes, easily - With little difficulty - With moderate difficulty - With extreme difficulty - No, impossible |

| **Question** | **Response** |
| --- | --- |
| 1. During the past 4 weeks have you been troubled by pain from your knee in bed at night? | - No nights - Only 1 or 2 nights - Some nights - Most nights - Every night |
| 1. During the past 4 weeks how much has pain from your knee interfered with your usual work (including housework)? | - Not at all - A little bit - Moderately - Greatly - Totally |
| 1. During the past 4 weeks have you felt that your knee might suddenly ‘give way’ or let you down? | - Rarely / never - Sometimes, or just at first - Often, not just at first - Most of the time - All of the time |
| 1. During the past 4 weeks could you do the household shopping on your own? | - Yes, easily - With little difficulty - With moderate difficulty - With extreme difficulty - No impossible |
| 1. During the past 4 weeks could you walk down one flight of stairs? | - Yes, easily - With little difficulty - With moderate difficulty - With extreme difficulty - No, impossible |
